# Seed Traits and Salt Tolerance Contribute to the Range Expansion of *Plantago coronopus* Along Winter‐Salted Roads in Central Europe

**DOI:** 10.1002/ece3.72406

**Published:** 2025-11-17

**Authors:** Henrietta Bak, Réka Fekete, Zsombor Miholcsa, Jenő Nagy, Sándor Jordán, Péter István Molnár, Attila Molnár V., Eszter Ruprecht

**Affiliations:** ^1^ HUN‐REN–UD Conservation Biology Research Group, Department of Botany University of Debrecen Debrecen Hungary; ^2^ Department of Botany University of Debrecen Debrecen Hungary; ^3^ Juhász‐Nagy Pál Doctoral School University of Debrecen Debrecen Hungary; ^4^ Hungarian Department of Biology and Ecology Babeş‐Bolyai University Cluj‐Napoca Romania; ^5^ Centre for Systems Biology, Biodiversity and Bioresources (3B) Babeș‐Bolyai University Cluj‐Napoca Romania; ^6^ Doctoral School of Animal Science University of Debrecen Debrecen Hungary

**Keywords:** adventive, biological invasion, dispersal, seed mass, seed shape, terminal velocity

## Abstract

Due to the expansion of road networks and the use of de‐icing salt, numerous salt‐tolerant plant species spread along roadsides worldwide. One of the most striking examples is a coastal halophyte, *Plantago coronopus* , which has been dispersing along road networks in Europe. Our aim was to investigate the role of seed dimorphism, seed traits of the two seed types (morphology, germination rate, wind dispersal ability), and salt tolerance in the successful roadside spread of *P. coronopus* . We compared the seed traits, salt tolerance, and the distribution along roadsides in Hungary of *P. coronopus* to four species of the *Plantago* genus native to Central Europe. We found that the native *P. lanceolata* was the most widespread along roadsides in Hungary, while *P. coronopus* was the third. Interestingly, *P. coronopus* was found almost exclusively along motorways. *P. coronopus* had almost twice as many large seeds as small seeds. Small seeds of this species had the lowest terminal velocity compared to the other species, which may contribute to the successful dispersal by wind and air currents caused by cars. Seeds of all the species germinated at 0.4% salt concentration, but with a reduced rate (except *P. major* ). Specimens from small seeds had higher shoot weight than those from large seeds and proved to have a higher reproductive potential even in salted soil. Our results show that seed dimorphism, through different dispersal capacity and germination success of the two seed types, as well as through different reproductive potential and salt tolerance of specimens developed from the two seed types, may contribute to the current rapid spread of *P. coronopus* along motorways in Hungary and Europe, and to its persistence under disturbed, spatially and temporally heterogeneous conditions.

## Graphical abstract

Our aim was to investigate the role of seed dimorphism and seed traits in the successful roadside spread of *P. coronopus* , and to compare its salt tolerance to other species of the *Plantago* genus native to Central Europe. Our results show that winter‐salted roadsides are suitable habitats for *P. coronopus* and other salt‐tolerant Plantago species. Seed dimorphism of *P. coronopus* contributes to its successful dispersal and adaptation to heterogeneous roadside conditions.

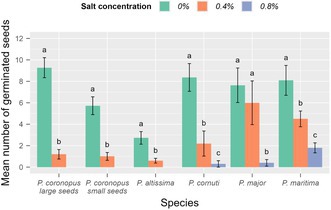

## 1 Introduction

Over the past decades, there has been a huge development of road networks worldwide (European Road Federation 2020). Several studies have shown that the density and traffic of road networks are related to the prevalence of alien and invasive plant species (Vilà and Pujadas 2001; Dark 2004). Roads are one of the main channels to conquer new habitats (Forman and Alexander 1998; Christen and Matlack 2009; Mortensen et al. 2009; Joly et al. 2011; Follak et al. 2018). A well‐known example is *Ambrosia artemisiifolia* L., a species native to North America that causes enormous agricultural damage and sickens many people with its pollen (Makra et al. 2004; Kazinczi et al. 2008). Several studies confirm that the expansion of road networks has contributed to the spread of this species in Europe, Asia, and Australia (Chauvel et al. 2006; Lavoie et al. 2007; Essl et al. 2009; Simard and Benoit 2010).

In addition, vehicles play an important role in the dispersal of a wide variety of plant propagules by creating air turbulence and transporting soil adhering to them (Strykstra et al. 1997; Zwaenepoel et al. 2006; Vitalos and Karrer 2009; von der Lippe et al. 2013).

Compared to the surrounding landscape, roadsides have different environmental parameters, and non‐native plant species may tolerate these stressful conditions more than native ones (Mack and Thompson 1982; Tyser and Worley 1992). As a result, roadside populations of alien and invasive plants may serve as a source of dispersal of certain species into surrounding natural habitats (Tyser and Worley 1992; Pauchard and Alaback 2004). Due to road construction and heavy vehicle traffic, compacted soil structure, altered water availability, and high concentrations of heavy metals, survival is difficult for many plant species (Hobbs 1991; D'Antonio and Vitousek 1992; Gelbard and Belnap 2003; Huber et al. 2016). Winter de‐icing salinization with rock salt (NaCl) increases the salt concentration in the soil (Norrström and Bergstedt 2001; Houska 2007; web1, web2). Åsteböl et al. (1996) and Norrström and Bergstedt (2001) also showed that leached salt accumulates in roadside soil within 10 m of the embankment. High salt concentrations cause osmotic stress in most plant species and also modify soil pH, nutrient availability, and soil permeability (Davison 1971). The high salt concentration has a significant effect on germination rate and speed, as well as on shoot and root elongation of plants (Bybordi 2010; Kaiwen et al. 2020).

However, the salt‐tolerant plant species can successfully colonize and spread along roadside areas due to the extensive use of winter de‐icing salt (Davison 1971). The spread of halotolerant species, especially coastal salt‐tolerant plant species along roadsides, is a common phenomenon (Scott and Davison 1982; Wróbel et al. 2006; Dítě and Dítětová 2016; Kocián et al. 2016; Hanselmann 2017). One of the best‐known roadside halotolerant plant species is *Plantago coronopus* L. (Fekete et al. 2021). This coastal plant species of the *Plantaginaceae* is usually annual, but it can be biennial or perennial under favorable conditions. It is native to coastal regions and islands of Europe and North Africa, and from the Black Sea to the Caspian Sea region of Tajikistan (Haeupler and Schönfelder 1989; Zając and Zając 2001; Villellas et al. 2013; Nikolić 2021). It is present as an introduced or adventive species in Australia, North, Central, and South America, some countries in Africa, and many countries across Europe (POWO 2024).

In the last four decades, the species is thought to have migrated from the Atlantic coast of Europe to Central Europe, including Hungary (Fekete et al. 2021). Newly documented populations suggest that the species is currently in an intensive phase of roadside dispersal in Europe (Schmidt 2021; Molnár V. et al. 2024).

Many other species of the *Plantago* genus are also able to withstand changes in habitat quality, cope with natural or anthropogenic disturbances (Sagar and Harper 1964; Hyland et al. 2010), and have been documented in disturbed habitats, such as roadsides. This is not surprising, given that many *Plantago* species have been observed to have high genetic diversity and to exhibit drought tolerance in terms of mucilages (Shahriari et al. 2018; Bagheri et al. 2022; Sharirari et al. 2024). Most commonly, the roadside appearance of *P. lanceolata* has been recorded (Drava et al. 2019; Abate et al. 2022), but *P. major* and *P. maritima* have also been documented in such ruderal habitats (Warwick and Briggs 1980; Scott and Davison 1985; Jaźwa and Klimek 2016; Baranov et al. 2021). In addition to *P. coronopus* , more than 20 other species of the genus are known to be salt‐tolerant, such as *P. cornuti*, *P. maritima* , and *P. media* (Ianovici 2011).

Many factors contributing to the success of invasive species have been identified (Alpert et al. 2000; Richardson and Pyšek 2006). For example, pre‐adapted traits that can help species survive in different environments (Callaway and Aschehoug 2000) and the ability of rapid evolutionary changes (Mack et al. 2000; Leger and Rice 2003; Parker et al. 2003). Other studies have identified high dispersal ability (Higgins et al. 1996), high reproductive capacity (Pyšek et al. 2004), rapid growth, and high competitive ability (Werner et al. 2010) as the most important traits of invasive species.

*P. coronopus* has a unique feature among the other *Plantago* species: seed dimorphism (Dowling 1933; Braza et al. 2010; Braza and García 2011), which is rare among plants (Ungar 1995; Khan and Gul 2006). All the seed pods of this species contain two types of seeds, which differ in size (Dowling 1933). Specimens developing from large seeds have a higher biomass than those developing from small seeds, which is also true during the vegetative and generative stages of development (Koelewijn 2004). Different types of seeds may also differ in their germination properties (Redondo‐Gómez et al. 2008). Seed dimorphism may play an important role in efficient seed dispersal and may represent an evolutionarily stable strategy in spatially or temporally heterogeneous habitats, such as salt marshes (Philipupillai and Ungar 1984; Khan and Ungar 1986; Geritz 1995). Therefore, it is not surprising that relatively many halotolerant species have dimorphic seeds (Song and Wang 2015; Yang et al. 2017). It has also been shown that the formation of dimorphic seeds, together with other plant traits (such as short‐livedness and high stature), contributes to the success of adventive species (Fenesi et al. 2019). Thus, this trait is expected to significantly contribute to the establishment and spread of range‐expanding species, such as invasive species.

Thereby, we hypothesize that besides salt tolerance, seed dimorphism and different seed traits of the two seed types help *P. coronopus* to colonize areas far from the native coastal zones, and to persist in salted, disturbed, constantly changing, and spatially heterogeneous environments. The objectives of our study were (1) to explore the distribution of *P. coronopus* and other species of the genus native to Central Europe along the national road network in Hungary, (2) to compare seed traits, and (3) to investigate the salt tolerance of *P. coronopus* together with that of some other *Plantago* species involved in our study as a comparison ( *P. altissima* , *P. cornuti*, *P. major* , and *P. maritima* ) through germination and plant growth experiments.

## 2 Material and Methods

### 2.1 Field Survey

**FIGURE 1:**
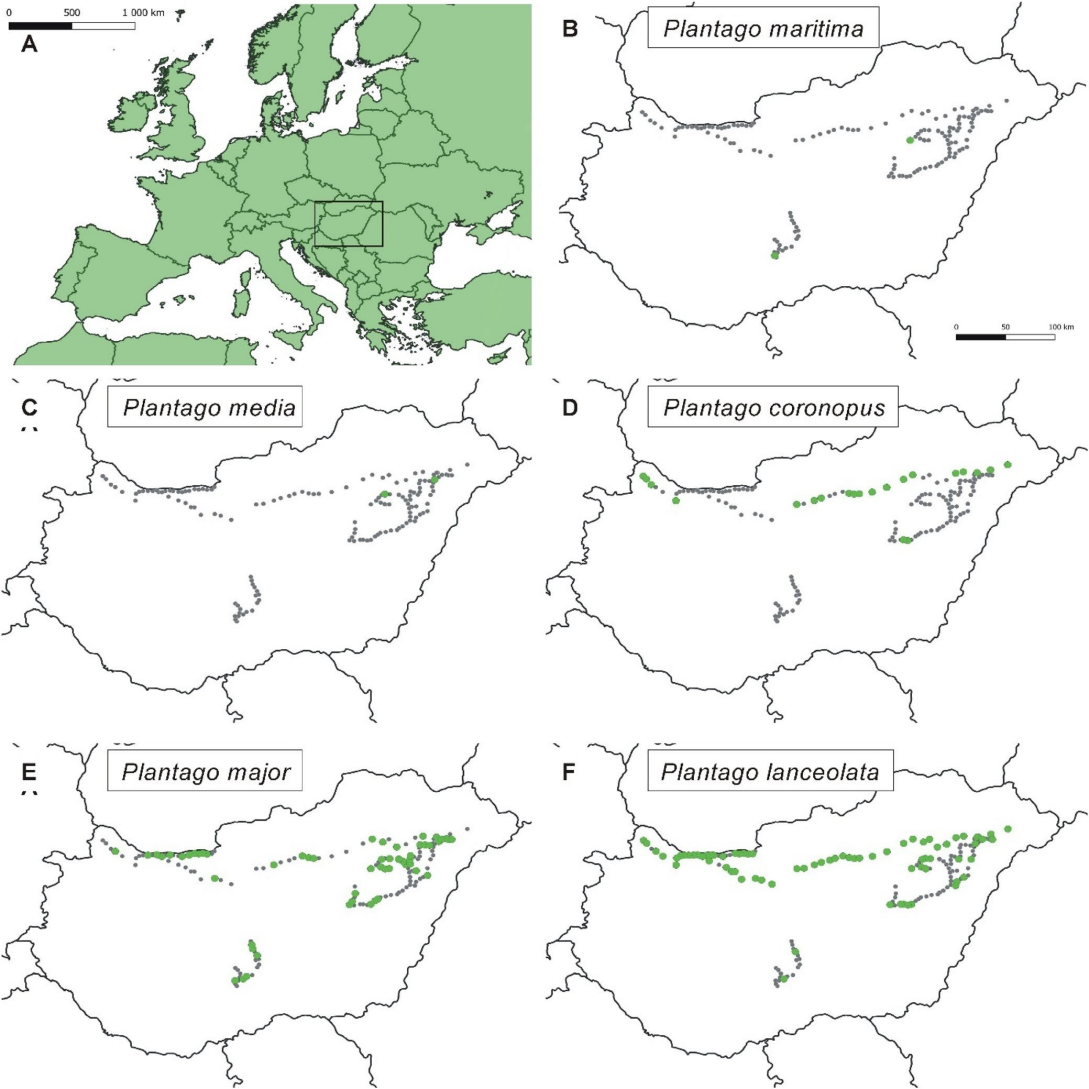
Occurrences of *Plantago* species found during roadside field surveys. The green points indicate where the species was found, and the gray points indicate where the species was not present.

The thematic road survey was conducted between May and July 2022 along randomly selected road sections of Hungary, with a preponderance in the northern part of the country, with a total of 152 sampling points (Figure 1).

The sampling points were located at a distance of about 5 km between each, where traffic conditions and rules allowed for stopping by car. For motorways, traffic rules allowed us to survey petrol stations and rest areas. At each sampling point, the road verge was surveyed in a 100 m long and 6 m wide strip, and at each point, the geocoordinates, altitude, and *Plantago* species with the number of specimens were recorded.

We used the online database of Hungarian Public Road Nonprofit Pte Ltd. Co. to categorize the surveyed roads as follows: motorways, highways (primary main roads, secondary main roads), and lower‐grade roads.

### 2.2 Seed Collection and Measurement of Seed Traits

Seeds of *P. altissima* , *P. cornuti*, *P. coronopus* , *P. major* , and *P. maritima* were collected in 2021 in Hungary and Romania, each species originating from two geographically distinct populations (Table 1). After field collection, the seeds were stored in paper bags at room temperature (ca. 20°C–23°C) until the start of the experiments. In August 2021, we estimated the proportion of small and large seeds per fruiting branch in *P. coronopus* by counting small and large seeds from a total of 200 fruiting branches of 49 specimens. From each fruiting branch, 10 capsules were randomly selected (or all if there were fewer than 10), and the number of small and large seeds developed per fruiting branch was counted. We examined the seeds from both populations of each species and seed type of *P. coronopus* .

**TABLE 1:** Seed collection sites and habitats for the studied *Plantago* species and populations (in superscript).

Species and population	Country	Settlement (county)	Habitat	Coordinates
*P. altissima* ^1^	Hungary	Kismaros (Pest)	Ruderal vegetation, abandoned field	47.82350°N; 18.99884°E
*P. altissima* ^2^	Hungary	Kunpeszér (Bács‐Kiskun)	Ruderal vegetation, abandoned field	47.09852°N; 19.26153°E
*P. cornuti* ^1^	Romania	Morişti (Cluj)	Salt grassland	46.78217°N; 23.76766°E
*P. cornuti* ^2^	Romania	Băile Someșeni (Cluj)	Salt grassland	46.77948°N; 23.6534°E
*Plantago coronopus* ^1^	Hungary	Veszprém (Veszprém)	Ruderal vegetation, road verge	47.10779°N; 17.86593°E
*Plantago coronopus* ^2^	Hungary	Káld (Vas)	Ruderal vegetation, road verge	47.09763°N; 17.06808°E
*P. major* ^1^	Hungary	Nagymaros (Pest)	Ruderal vegetation, road verge	47.82632°N; 18.97270°E
*P. major* ^2^	Romania	Inucu (Cluj)	Ruderal vegetation, abandoned field	46.84160°N; 23.24882°E
*P. maritima* ^1^	Hungary	Agárd (Fejér)	Ruderal vegetation, city park	47.20049°N; 18.60729°E
*P. maritima* ^2^	Romania	Băile Someșeni (Cluj)	Salt grassland	46.77948°N; 23.65345°E

On 7 April 2022, following the method of Török et al. (2013), we determined the thousand‐seed weight by population of each species using an analytical balance, that is, we weighed 3 batches of 100 seeds. In the case of *P. coronopus* , we measured the thousand‐seed weight of small and large seeds separately. The values obtained were averaged by species and, in the case of *P. coronopus* , by seed type.

In March 2022, using 50–50 seeds of each of the five *Plantago* species studied, and by treating the small and large seeds separately for *P. coronopus* , we determined the seed shape of each species. The seeds were photographed using DinoCapture 2.0, and the length, width, and height of the seeds were measured using ImageJ. The measured values were used to calculate the seed shape (LEDA Traitbase 2022; https://uol.de/en/landeco/research/leda/standards) by the formula:

$$V_{s}=\Sigma {\left(x_{i}-\text{mean}\;\left(x\right)\right)}^{2}/n.$$

n=3,

$$x_{1}=\text{length}/\text{length},\;\;x_{2}=\text{height}/\text{length},\;x_{3}=\text{width}/\text{length}.$$

The seed shape variance index can range from 0 to 0.33. The closer the value is to 0, the rounder the seed shape is, and larger values indicate a long or flat seed shape (Kleyer et al. 2008).

### 2.3 Seed Dispersal Test

The wind dispersal ability (anemochory) of the seeds of the studied *Plantago* species was estimated by measuring seed terminal velocity, that is, the maximum rate of fall, calculated by dividing the height of the fall by the duration of seed descent (Andersen 1992) with an instrument we built. The duration of seed fall was measured in a 1.47 m long and 20 cm diameter vertical PVC tube. The tube restricted the air movement in the room, so the seeds descended in the tube by free fall only. We recorded the experiments using a Canon 60D camera (NTSC, 60 frame/s resolution). The recordings were evaluated using Adobe Premiere Pro 2020 software. The number of frames recorded during the seed fall was converted to seconds to express the duration of the fall. The experiment was conducted with 50–50 seeds per species, with small and large seeds of *P. coronopus* being tested separately.

### 2.4 Soil Sample Collection and Analysis of Salt Content

In March 2019, soil samples were collected at randomly selected sampling sites (*n* = 66) along motorways (*n* = 22), highways (*n* = 26), and lower‐grade roads (*n* = 18). At each sampling site, soil samples were collected at three distances (0.1, 0.5, and 1 m; *n* = 198 in total) from the roadside. Samples were collected from a soil depth of 0.1 m, resulting in 0.1 × 0.1 × 0.1 m soil cubes. Samples were placed in paper bags and stored at room temperature until analysis. The salt content analysis of the samples was performed by the University of Agricultural and Life Sciences Research Institute in Karcag (Hungary) in July 2019.

### 2.5 Testing Salt Tolerance by a Germination Experiment

Between 4 May and 6 June 2022, the salt tolerance of the *Plantago* species was tested by a germination experiment. Germination was carried out in Petri dishes on filter paper. Each Petri dish contained 20 seeds. The filter paper was moistened with 5 mL of the solution. The solutions were prepared from distilled water and pure NaCl. In total, four different solutions were used: a control containing 0 m/m% salt and three different NaCl concentrations: 0.4 m/m% (68.45 mM, 6.84 dS/m), 0.8 m/m% (136.92 mM, 13.69 dS/m), and 1.2 m/m% (205.34 mM, 20.53 dS/m). Five replicates per concentration were prepared for each population and seed type. Our salt treatments were chosen to correspond to the salt content found in our field sampling sites and to the salinity categories of the Food and Agriculture Organization (FAO) classification. Germination was carried out in a climate chamber (Sanyo MLR 352, Sanyo/Panasonic Healthcare Co. Ltd., Japan) at 20°C with alternating periods of 12 h light (250 μmol m^−2^ s^−1^) and 12 h darkness in the Laboratory of the University Botanical Garden of Cluj‐Napoca. The Petri dishes were checked every 2–3 days, and new seedlings were recorded and removed. The number of germinated and non‐germinated seeds was expressed for each sample at the end of the experiment.

### 2.6 Testing Salt Tolerance by a Plant Growth Experiment

In the summer of 2022, we studied the effects of four different NaCl concentrations on the development of young plant specimens in the outdoor experimental site of the “Alexandru Borza” Botanical Garden in Cluj‐Napoca. The experimental site had a transparent polyethylene roof, where plants were not exposed to precipitation, and was surrounded by wire mesh for protection. The experiment was therefore conducted under natural light conditions. A total of twenty 1 L pots per species and, in the case of *P. coronopus* , per seed type (*n* = 6), were used during the experiment. 30 seeds were sown within each pot filled with horticultural potting soil. The soil was left to rest for 2 years before starting the experiment. The pots were stored on tray‐less tables and watered with tap water as needed during the experiment to avoid drying out. After sowing, 0.025 g of nutrient mixture (20% P_2_O_5_, 44% K_2_O, 0.05% Fe, 0.025% Mn, 0.008% Zn, 0.006% Cu, 0.02% B, 0.0035% Mo) and 0.05 g of nitrogen in 30 mL of distilled water were added to each pot. The seedlings were continuously thinned until the seeds of all species had germinated; thus, the seedlings were of the same age at the time of treatment application. Finally, one seedling was left in each pot for the growth experiment.

The seedlings were left to grow for 7 days (from 3 June to 10 June 2022), and after that they were watered with distilled water or NaCl solution. Four different concentrations of NaCl solutions were used, similar to the germination experiment, that is, 0 m/m% as control, 0.4, 0.8, and 1.2 m/m% NaCl. We calculated the water quantity necessary for attaining field capacity in our 1 L experimental pots. NaCl concentrations were adjusted to this water quantity (30 mL). We used 5 samples per salt concentration (*n* = 4) for each species and seed type (*n* = 6), thus having a total of 120 pots in our growth experiment. To avoid osmotic shock, the NaCl solutions were applied to the pots in two batches (on 10 and 17 June), each with 30 mL of distilled water or NaCl solution. The pots were checked seven times during the study, and the number of dead specimens was recorded each time. The last check was carried out on 22 July 2022, when we counted the number of inflorescences of each specimen that survived the treatments. After this, specimens were removed, their roots were washed free of soil, and the plants were dried in an oven at 60°C for 72 h. After drying, roots and shoots were weighed separately using an analytical scale.

### 2.7 Data Analysis

#### 2.7.1 Seed Dispersal Test

First, we checked the distribution of terminal velocity values and applied ordered quantile normalizing transformation (“bestNormalize” package, Peterson and Cavanaugh 2020, Peterson 2021) to approximate normal distribution (*n* = 300, Shapiro–Wilk's test: *W* = 1, *p* = 0.91; Shapiro and Wilk 1965). Then, we performed linear regression (LM) and post hoc test by Tukey's adjustment (“emmeans” package, Lenth 2023) to compare terminal velocity of the species.

#### 2.7.2 Salt Content of Soil Samples

To compare the salt content of soil samples collected along different road types and at different distance categories, we applied a simple LM with log‐transformed salt concentration as the response variable and road type and distance category as predictors. We performed a post hoc test by Tukey's adjustment to compare salt concentration per road type and distance category.

#### 2.7.3 The Effect of NaCl Concentrations on the Success of Seed Germination and Development of Young *Plantago* Specimens

We used the number of germinated seeds as a response variable in a negative binomial generalized linear mixed effects regression (GLMM), including species and NaCl concentration as fixed effects and population (Table 1) as a random effect (“glmmTMB” package, Brooks et al. 2017). Then, we applied post hoc tests by Tukey's adjustment to compare the effects of salt concentrations and species on germination success.

We evaluated separate LMs using data on shoot weight (log‐transformed) and root weight (square‐root‐transformed) as response variables. Finally, we performed a Poisson GLM on the number of inflorescences, keeping only records with > 0 values to optimize model performance. We included NaCl concentration and species as predictors in each model described above. We applied post hoc tests by Tukey's adjustment to compare the effects of salt concentrations and species on the above traits. Since there was no detectable growth at the highest NaCl concentration, we excluded those observations from the models.

All data analyses were performed in R v4.2.1 (R Core Team 2022), including the above‐mentioned packages.

## 3 Results

### 3.1 Distribution of *Plantago coronopus* and Other *Plantago* Species Along Roadsides in Hungary

Of the 152 roadside points surveyed, 39 were located along motorways, 55 along highways, and 58 along lower‐grade roads. A total of five species of the genus *Plantago* were found during the surveys; these were *P. coronopus* , *P. lanceolata* , *P. major* , *P. maritima* , and *P. media* (Figure 1). *P. lanceolata* was the most frequent of all the species in roadsides, with *P. coronopus* being third in this order. These two species were typically found along motorways, while *P. major* , the second most frequent species, occurred particularly along lower‐grade roads (Table 2). The presence of *P. maritima* and *P. media* along roadsides was occasional. *P. coronopus* and *P. lanceolata* were found in huge numbers at surveyed points along motorways, while *P. major* was present with fewer specimens along all three road types. *P. coronopus* was found only at two highway survey points, and it was absent from the verges of lower‐grade roads (Table 2).

**TABLE 2:** Number of occurrences, frequency and average number of specimens ± SD of *Plantago coronopus* and other *Plantago* species found along roadsides in Hungary by road type.

Species	Number of occurrences	Frequency (%)	Average number of specimens ± SD
*Plantago coronopus*			
Motorway	19	48.72	920 ± 1609
Highway	2	3.64	1
Lower‐grade road	0	0	0
Total	21	13.82	
*P. lanceolata*			
Motorway	34	87.18	867 ± 1016
Highway	29	52.73	98 ± 107
Lower‐grade road	14	24.14	20 ± 26
Total	77	50.66	
*P. major*			
Motorway	12	30.77	15 ± 19
Highway	21	38.18	21 ± 51
Lower‐grade road	29	50	38 ± 52
Total	62	40.79	
*P. maritima*			
Motorway	0	0	0
Highway	1	1.82	1
Lower‐grade road	1	1.72	1
Total	2	1.32	
*P. media*			
Motorway	0	0	0
Highway	0	0	0
Lower‐grade road	3	5.17	1
Total	3	1.97	

### 3.2 Seed Traits of *Plantago coronopus* and Other *Plantago* Species

Based on our estimation, on average, 1.79 (±1.40 SD) large and 0.78 (±1.26 SD) small seeds were formed within a fruiting branch of a *P. coronopus* specimen, thus almost twice as many large as small seeds. All branches, regardless of whether they produced any seeds or not, were included in the calculation, which represents the overall reproductive output of a specimen.

The small seeds of *P. coronopus* had the lowest thousand‐seed weight and the large seeds had the second lowest value, while *P. altissima* had by far the highest thousand‐seed weight of all the studied species. Furthermore, we found that the large seeds of *P. coronopus* had 2.5 times higher thousand‐seed weight than its small seeds (Table 3). Concerning seed shape, we found that, on average, the large seeds of *P. coronopus* were the roundest, while the seeds of *P. cornuti* were the least round, being the longest ones (Table 3).

**TABLE 3:** Thousand‐seed weight (*n* = 300) and average seed shape index (*n* = 50) of *Plantago coronopus* seed types and other *Plantago* species.

Species	*P. coronopus* large seeds	*P. coronopus* small seeds	*P. altissima*	*P. cornuti*	*P. major*	*P. maritima*
Thousand‐seed weight (g)	0.116	0.047	1.456	0.892	0.232	0.426
Seed shape	0.058	0.071	0.080	0.104	0.077	0.084

*Note:* Seed shape is a measure of variance, ranging from 0 to 0.33. The closer the value is to 0, the rounder the seed; the closer to 0.33, the flatter or longer the seed (Kleyer et al. 2008).

### 3.3 The Wind Dispersal Ability of *P. coronopus* and Other *Plantago* Species

**FIGURE 2:**
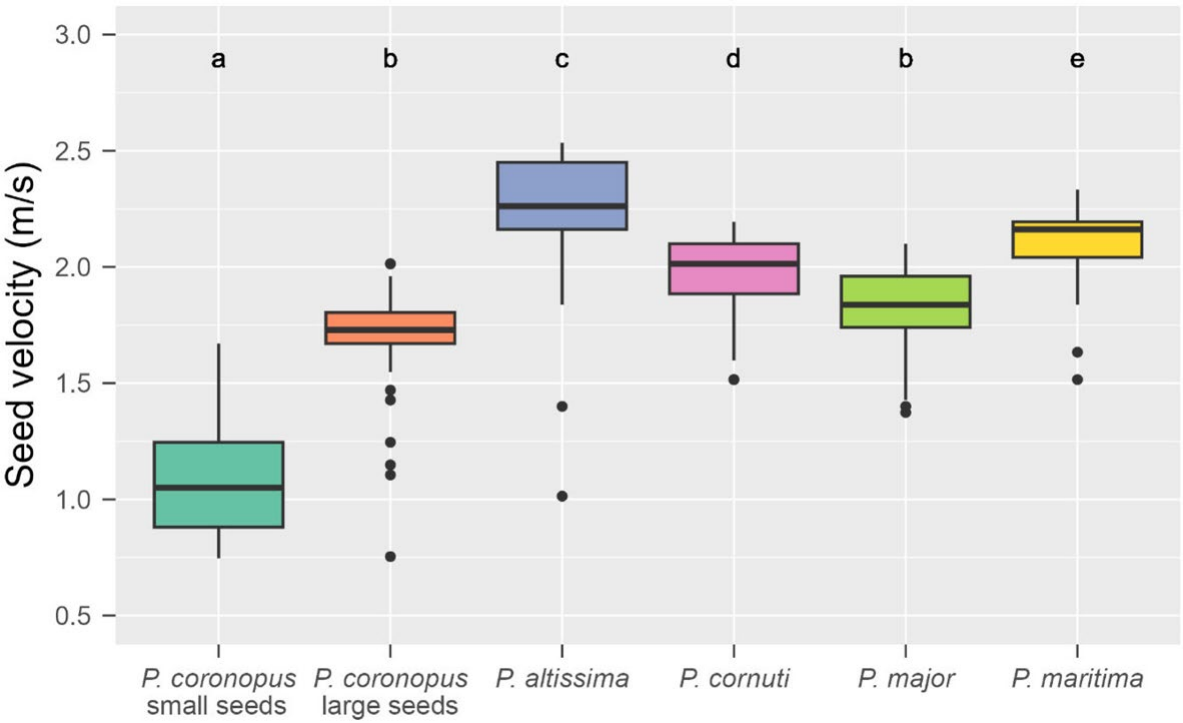
Differences in seed terminal velocity between *Plantago coronopus* seed types and other *Plantago* species (*n* = 50). Boxes represent values between the 1st and 3rd quartiles, whiskers represent values outside the interquartile range, and dots represent outlier values. The horizontal line in the box shows the median. Letters above the boxes denote significant differences at *p* < 0.05.

Seed terminal velocity differed between the studied *Plantago* species and *P. coronopus* seed types (*R* ^2^ = 0.66, *F*_5294_ = 115.9, *p* < 0.001). The terminal velocity of small seeds of *P. coronopus* was found to be significantly lower than that of large seeds and significantly lower than that of seeds of all the other species. This indicates the high ability of small seeds to be dispersed by wind. Seeds of *P. altissima* had the highest terminal velocity of all the species studied (Figure 2).

### 3.4 Salt Content of Soil in Roadsides

We found differences in the salt content of soil with distance to the roadside but not between road categories (*R* ^2^ = 0.05, *F*_4193_ = 3.5, *p* = 0.008, Table 4). Throughout all three road categories, the salt content of the soil was the highest close (0.1 m) to the roadside and lowest at a 1 m distance from the roadside (Table 4). A 0.4% salt concentration was typical only for highways and lower‐grade roads, and only very close to the roadside (at a 0.1 m distance). However, maximum salt concentration values exceeded 1% in the case of all three road types and distance categories.

**TABLE 4:** Salt concentration (mean ± SD) of soil samples per road type and distance category.

Road type	Distance category		
	0.1 m^A^	0.5 m^AB^	1 m^B^
Motorway (*n* = 22)^A^	0.194 ± 0.143^A^	0.156 ± 0.086^AB^	0.135 ± 0.079^B^
Highway (*n* = 26) ^A^	0.375 ± 0.446^A^	0.223 ± 0.200^AB^	0.149 ± 0.178^B^
Lower‐grade road (*n* = 18) ^A^	0.485 ± 0.504^A^	0.247 ± 0.350^AB^	0.148 ± 0.205^B^

*Note:* Letters in superscript show statistically significant (*p* < 0.05) differences between road types based on Tukey's post hoc tests.

### 3.5 Salt Tolerance During Germination of *Plantago coronopus* and Other *Plantago* Species

**FIGURE 3:**
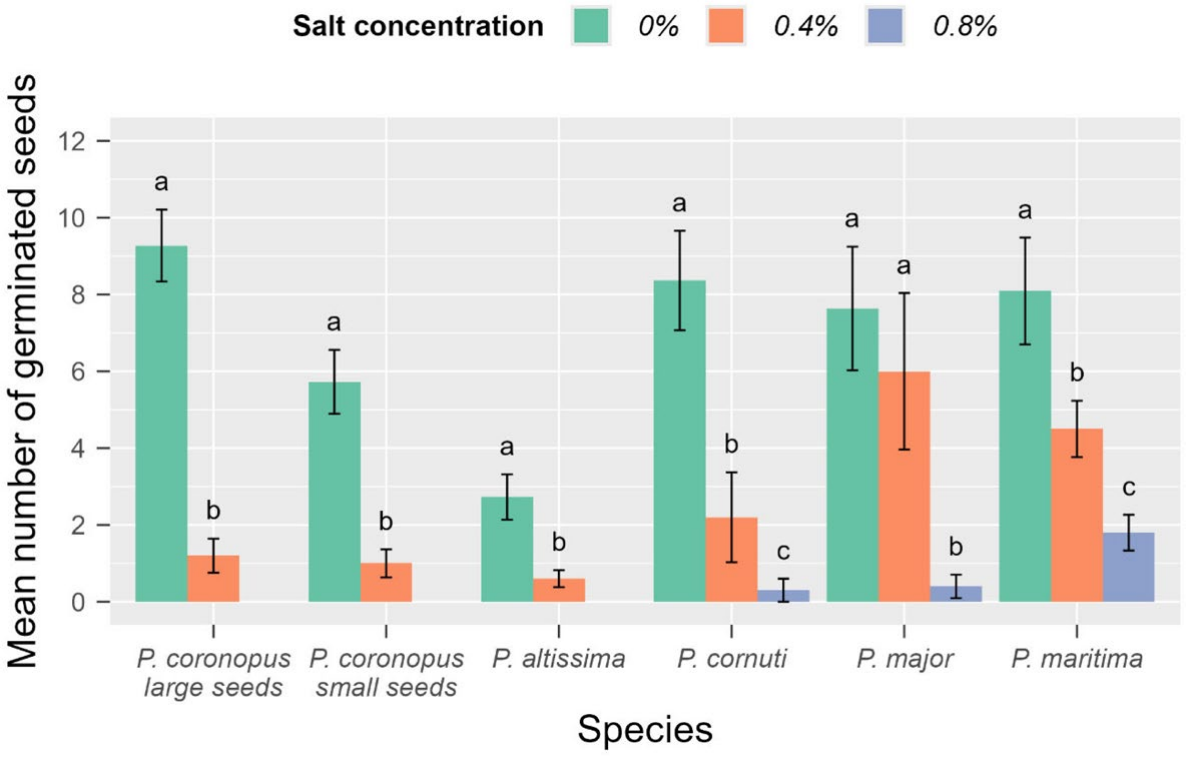
Number of seeds germinated in the control and two lower salt (NaCl) concentrations per species and *Plantago coronopus* seed types. 20 seeds were put to germinate per sample (Petri dish). Columns with vertical lines represent mean ± SE; letters above columns denote significant differences between different salt concentrations at *p* < 0.05.

**FIGURE 4:**
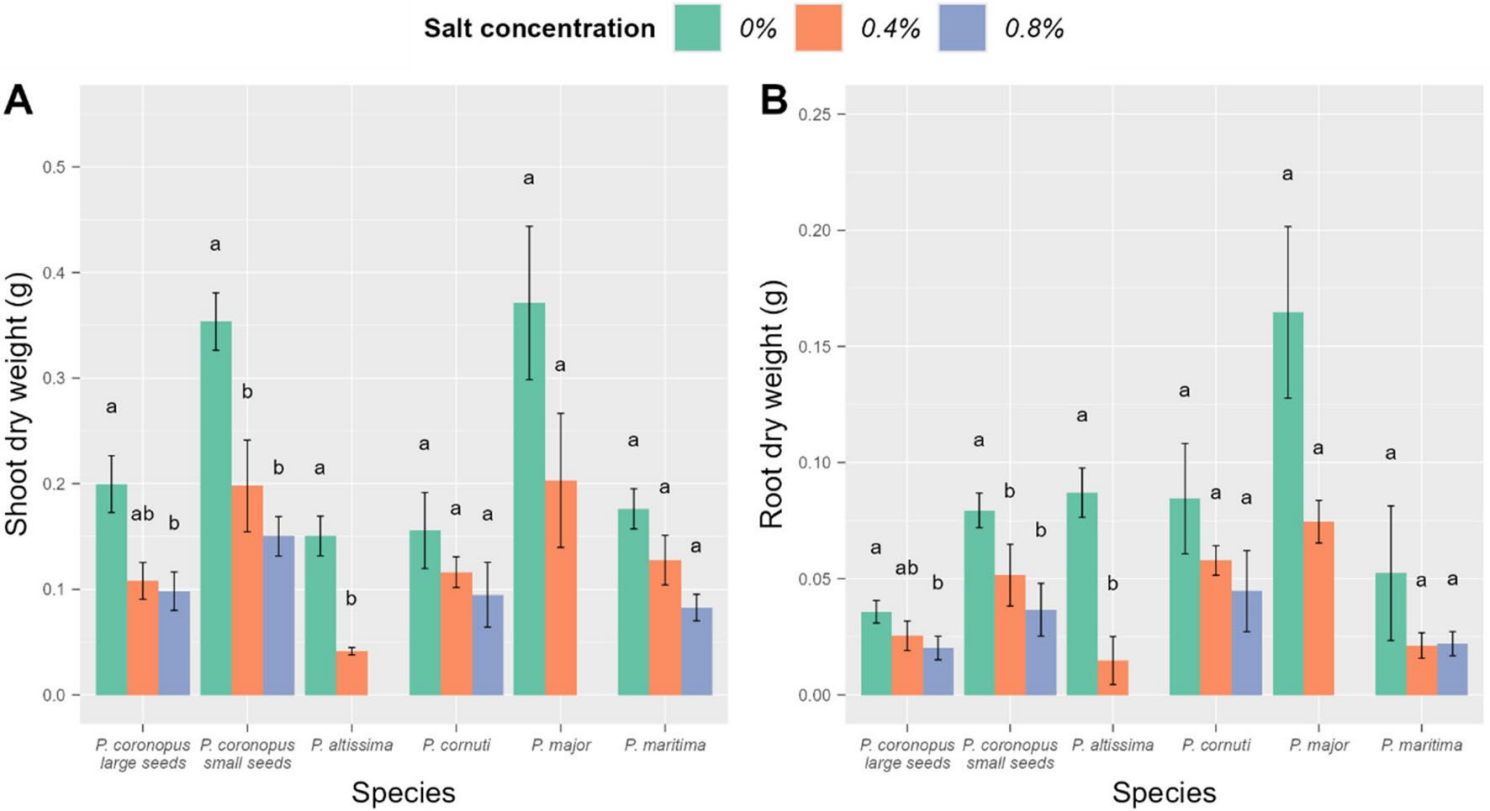
Comparison of shoot and root weight values of *Plantago coronopus* and other *Plantago* species between the control and two lower salt concentrations (*n* = 5 per species and seed type for each salt concentration). Columns with vertical lines represent mean ± SE, letters above columns denote significant differences between different salt concentrations at *p* < 0.05.

Salt concentration significantly affected seed germination, and there were differences between species in respect of salt tolerance during germination (Table S1). Seeds of all species germinated at both the control and the lowest (0.4%) NaCl concentration. For all species and seed types, the germination counts observed in the control were significantly higher than those observed at 0.4% and 0.8% NaCl concentrations, except *P. major* (Figure 3). The highest germination success of small and large *P. coronopus* seeds was observed in the control; germination success was lower at the lowest salt concentration, and there was no germination at all under NaCl concentrations higher than 0.4% (Figure 3). There was no significant difference in the salt tolerance during germination between small and large seeds of *P. coronopus* , but large seeds germinated at a higher rate (51%) than small seeds (31%; Figure 4) on the salt‐free control medium. *P. maritima* was the only species that germinated at all four salt concentrations, including the highest (1.2%) one.

### 3.6 Effect of Salt on the Development of Young *Plantago coronopus* and Other *Plantago* Specimens

In the growth experiment, we found that for all *Plantago* species and seed types, the survival rate of specimens in control pots was 100%, and with increasing NaCl concentrations, survival decreased (Table 5). Grown specimens of *P. altissima* and *P. major* did not survive at higher salt concentrations (above 0.8%), whereas 60%–80% of the specimens of *P. coronopus* (from both seed types), *P. cornuti*, and *P. maritima* survived even at a 0.8% salt concentration. We have to note that the survival of *P. coronopus* specimens developed from large seeds was 100% even at this high salt concentration. Only specimens of *P. maritima* were able to survive, albeit at low rates, at the highest (1.2%) salt concentration.

**TABLE 5:** Survival until the end of the experiment of *Plantago coronopus* and other *Plantago* specimens grown in soils with different salt (NaCl) concentrations.

NaCl concentration	Number of survived specimens/total number of specimens					
	*Plantago coronopus* large seeds	*Plantago coronopus* small seeds	*P. altissima*	*P. cornuti*	*P. major*	*P. maritima*
0%	5/5	5/5	5/5	5/5	5/5	5/5
0.4%	5/5	5/5	2/5	5/5	2/5	5/5
0.8%	5/5	4/5	0/5	4/5	0/5	3/5
1.2%	0/5	0/5	0/5	0/5	0/5	1/5

Growing on saline soil not only affected the survival of the specimens but also their shoot and root weight; however, the response of species was not uniform (Tables S2 and S3). In the control (no salt applied), *P. coronopus* specimens from small seeds had higher shoot weight than specimens from large seeds (Table S2). Shoot and root weight of *P*. *coronopus* specimens from small seeds and of *P. altissima* were lower when grown at the lowest salt concentration (0.4% NaCl), compared to the control (Figure 4). At a higher salt concentration (0.8% NaCl), in addition, *P. coronopus* specimens from large seeds and *P. major* were also affected. Two of the studied *Plantago* species (*P. cornuti* and *P. maritima* ) tolerated not only the 0.4% but the 0.8% salt concentration, and their shoot and root weight in salted pots did not differ from those in the control (Figure 4).

Flowering was observed in only two species, *P. major* and *P. coronopus* , during the growth experiment. Specimens of *P. major* and *P. coronopus* from large seeds developed inflorescences only in control pots, whereas specimens of *P. coronopus* from small seeds were able to develop inflorescences at a 0.4% and 0.8% NaCl concentration in addition to the control (Tables 6 and S4).

**TABLE 6:** Average number and SD of inflorescences and number of flowering individuals of *Plantago coronopus* and *P. major* at different NaCl concentrations.

NaCl concentration	*Plantago coronopus* large seeds^AB^	*Plantago coronopus* small seeds^A^	*P. major* ^B^
	Average number ± SD of inflorescences/number of flowering individuals		
0%	3.5 ± 0.7/2	5.4 ± 1.1/5	1.5 ± 1/4
0.4%	0	6/2	0
0.8%	0	5/1	0

*Note:* Letters in superscript show statistically significant (*p* < 0.05) differences between *P. coronopus* and *P. major* seeds based on Tukey's post hoc tests.

## 4 Discussion

Several species of the *Plantago* genus are reported to be common along roadsides due to their ruderal strategy (Roberts and Boddrell 1984). In our study, we showed that Hungarian roadsides are suitable habitats for the range‐expanding *P. coronopus* and several native *Plantago* species, as we found *P. coronopus*, *P. lanceolata* , and *P. major* to be very frequently appearing along surveyed roads, while *P. maritima* and *P. media* were only occasionally present at very few sampling points.

### 4.1 Distribution of *Plantago coronopus* and Other *Plantago* Species Along Roads

The high salt tolerance of halotolerant species allows them to colonize and spread in roadside areas maintained by salting in winter (Gerstberger 2001; Wróbel et al. 2006; Šerá 2008). Halotolerance is a plant adaptation to environmental stress, and stress‐tolerant species are weak competitors, thus occurring in habitats with low vegetation cover and competitive pressure.

In Hungary, continental saline habitats are widespread, especially in the eastern part of the country (Tóth 2010). Thus, it is not surprising that besides *Plantago* species, several other salt‐tolerant species have been found in roadside habitats of the country, the most common being *Puccinellia distans* , *Festuca pseudovina*, *Podospermum canum*, and *Hordeum hystrix* (Fekete et al. 2021, 2022). This also suggests that the saline soils of road verges are suitable habitats for halotolerant species. As roadsides are also highly disturbed habitats, it is likely that species that are both salt‐tolerant and have a ruderal strategy (Grime's RS strategy; Grime 1977) may be highly successful in these artificial habitats. Truscott et al. (2005) showed that the occurrence of ruderal plant species is higher along roadsides with higher traffic.

Interestingly, *P. coronopus* was almost exclusively found along motorway verges. This result supports that the species may have reached Hungary via the international motorway network, with the first occurrences recorded in the western part of the country (Schmidt et al. 2016), suggesting that the first populations of the species may have arrived from Western Europe. Fekete et al. (2021) also pointed out that the species was able to conquer the inland areas of the continent from its native coastal range thanks to the international motorway network. Our results suggest that currently, *P. coronopus* does not have the potential to expand from motorways to highways and lower‐grade roads despite its presence in large numbers along motorways. Whether it is because of a lag phase in its future spread toward lower‐grade roads throughout the country or because its dispersal has other obstacles needs further investigation.

The new populations found in Hungary in 2022 and recent data about its occurrence in Bosnia and Herzegovina, Bulgaria, Romania, and the Czech Republic (Nugent 2018; Schmidt and Maslo 2020; Schmidt 2021; Křenová et al. 2021) indicate that the species spreads further toward the central and eastern parts of Europe. The rapid range expansion of this species in salted roadsides suggests that it is adapted to changing environmental conditions caused by the high traffic and road maintenance disturbances (Forman and Alexander 1998; Tyser et al. 1998; Benefield et al. 1999; Trombulak and Frissell 2000).

Our survey showed that the native *P. lanceolata* was also very frequent along motorways (found in 87% of motorway sampling points), suggesting that this species also successfully inhabits motorway verges, and that its dispersal within its native range is probably facilitated by roads as artificial corridors. *P. major* was also frequently found along roads, being most common along lower‐grade roads and least common along motorways.

### 4.2 Contribution of Seed Traits to the Dispersal Success of *Plantago coronopus* Along Roads

Several studies have shown that low seed mass is a dominant functional trait in plant dispersal, often associated with high seed production, a persistent seed bank, and efficient wind dispersal (Bekker et al. 1998; Westoby et al. 1996). The seed dimorphism may also influence the intra‐ and inter‐annual dynamics of soil seed banks (Philipupillai and Ungar 1984; Venable and Levin 1985; Wertis and Ungar 1986; Venable et al. 1995). Thus, the persistent seed bank plays an important role in the long‐term persistence of the species in a given area and efficient spread over time (Mandák and Pyšek 2001; Atia et al. 2011; Čalasan and Kadereit 2023).

For anemochorous plant species, the median dispersal distance is determined by seed release height, average horizontal wind speed, and velocity (Greene and Johnson 1989). Velocity is strongly correlated with wind load, that is, the square root of seed weight/seed area, and can be used as an indicator of seed dispersal ability (Augspurger and Franson 1987). The thousand‐seed weight of large seeds of *P. coronopus* was 2.5 times higher than that of small seeds. The large seeds were rounder, but the velocity of the small seeds was significantly lower than that of the large seeds and lower than that of all the other species studied. The velocity of large seeds was also significantly lower compared to all other species studied. These results suggest that low terminal velocity, thus high potential for wind dispersal, very probably plays an important role in the successful roadside dispersal of *P. coronopus* , as it allows its propagules to remain longer in the air than those of other *Plantago* species, and thus, to make better use of wind and vehicle‐generated air currents. A comparison of the two types of seeds also shows that small seeds are more efficient than large seeds at dispersing by wind and different air currents. In addition, small seeds may also be able to form a more persistent seed bank than large seeds, as it has been described previously for other species with dimorphic seeds (Philipupillai and Ungar 1984). Seed dimorphism is considered an evolutionarily stable strategy in spatially or temporally heterogeneous habitats, such as saline habitats (Philipupillai and Ungar 1984; Khan and Ungar 1986; Geritz 1995).

### 4.3 The Role of Salt Tolerance in the Roadside Spread of *Plantago coronopus*

Winter de‐icing salt with NaCl has several negative effects on the environment and vegetation, including contamination of surface water, drinking water, and groundwater, and corrosion of vehicles and bridges (Amrhein et al. 1992). Increased salt concentrations in soils can damage the root system and photosynthetic function of leaves, alter pH and nutrient availability, cause osmotic stress, thereby altering vegetation composition, and promote the spread of stress‐tolerant halotolerant (salt‐tolerant) plants (Davison 1971; Kaiwen et al. 2020). The lowest salt concentration, 0.4%, which we used, is considered to be a medium salt concentration by the FAO, which already inhibits the germination of seeds and development of many plant species. It is, therefore, interesting to note that seeds of all *Plantago* species we tested were able to germinate (albeit most of them at a lower rate than the control) at the 0.4% salt concentration, with a high survival rate of the seedlings. The survival of specimens of several species (*P. cornuti*, *P. coronopus* , and *P. maritima* ) was high even at a higher salt concentration (0.8%). The remarkable salt tolerance of *P. major* seeds during germination is surprising since this species has not been categorized as typically salt tolerant (Ellenberg S 0/9).

Although the salt tolerance of *P. coronopus* during germination was found to be medium in comparison to the other studied *Plantago* species, both its large and small seeds were able to germinate at a medium salt concentration (0.4 m/m%) and its seedlings survived at a much higher salt concentration (0.8 m/m%) than the average salt concentrations of road verges found in the analysis of soil samples.

No difference was found in the salt tolerance of the two seed types of *P. coronopus* , but a significant difference was observed in the control concentration without salt: the germination rate of large seeds (51%) was much higher than that of small seeds (31%). In the dimorphic *Tragopogon pratensis* subsp. *pratensis*, it was also observed that the germination rate of large seeds was higher than that of small seeds, and that the germination success of dimorphic species is mainly influenced by seed size rather than seed morphology (van Mölken et al. 2005).

Surprisingly, smaller *P. coronopus* seeds resulted in larger specimens, which produced more inflorescences. This contradicts the results of previous studies on other species having dimorphic seeds (Ellison 1987; Zhang 1993; Koelewijn 2004). In addition, while specimens from large seeds were more tolerant to salted growing conditions in respect of shoot and root weight, only specimens from small seeds were able to develop inflorescences on saline soil. This indicates that the reproductive capacity of *P. coronopus* specimens from small seeds is higher compared to specimens from large seeds under salt‐free conditions, but also in soils containing salt. Thus, our results point out that, besides lower seed velocity, and thus higher potential for wind dispersal of small seeds and higher germination success of large seeds, seed dimorphism plays a role in the salt tolerance and reproductive potential of *P. coronopus* as well. By this means, seed dimorphism may contribute to the rapid spread of *P. coronopus* along winter‐salted motorways in Hungary and Europe, as well as to its persistence under disturbed, spatially and temporally heterogeneous conditions.

The life history strategy of *P. coronopus* differs from the other *Plantago* species involved in our study, as *P. coronopus* can have an annual, biennial, or perennial life history, while the other species are all annuals (Király 2009; Király et al. 2011). The perennial life history may be advantageous in stable environmental conditions, as it allows the persistence of plant specimens in time (Ning et al. 2022). In the case of biennial or perennial life histories, adaptation to heterogeneous environments may be more successful and the reproductive rate higher due to the short generation time (González‐Paleo et al. 2019; Ning et al. 2022). Our results suggest that all the *Plantago* species studied may be suitable for dispersal through road networks, even in road verges maintained by salting. So far, apart from *P. coronopus* , *P. lanceolata* and *P. major* have proven their successful presence along roadsides, as our survey showed that they were present with high frequency, especially along motorways (except *P. major* ). The high roadside presence of *P. coronopus* may also be explained by its combined CSR strategy, that is, stress tolerance, competitive ability, and disturbance tolerance (Grime 1977). It is possible that in the future, besides the adventive *P. coronopus* , the native *P. lanceolata* and *P. major* , and other *Plantago* species may also become common along salt‐maintained roads, as they slightly tolerate medium salt concentrations during their germination and plant growth life stages. Furthermore, their high genetic diversity and drought tolerance in terms of mucilages may be largely responsible for the successful spread of these species (Shahriari et al. 2018; Bagheri et al. 2022; Sharirari et al. 2024). The spread of *P. coronopus* in Hungary has an increasing rate, but the species has not yet spread from the motorway verges to highways and lower‐grade roads. The highways and lower‐grade roads are less isolated from the surrounding landscape, and their verges form more of a natural barrier, unlike the verges of motorways. It may only be a matter of time before the species moves from the more closed verges of motorways to the verges that form a more natural transition to the surrounding landscape. This would mean the escape of this adventive species from ruderal into more natural saline habitats.

Based on our results, we can say that there are several factors besides the life history strategy that may be responsible for the success of *P. coronopus* . One of the key factors seems to be the low seed weight of small and large seeds, which allows especially small seeds, but also large ones, to be well dispersed by wind and different air currents. The high germination vigor of large seeds, as well as the successful development and high reproductive output of specimens from small seeds, may also be important.

The intensive spread of *P. coronopus* is of concern for several reasons, as the pollen of this species is allergenic (Iglesias‐Otero et al. 2015), and it has been shown to be able to invade agricultural land as a weed (Cirujeda et al. 2011). Due to the large extent of natural saline habitats in Hungary, the spread of the salt‐tolerant *P. coronopus* is particularly important to monitor, as several cases have shown that non‐native species are able to colonize natural habitats (Barbosa et al. 2010; Pollnac et al. 2012; McDougall et al. 2018), potentially reducing biodiversity (Van der Putten et al. 2010). In regions where similar habitats to those occurring in the species' natural range are found (e.g., European salt marshes), the potential for invasion should be considered. For roads crossing such habitats, the timing of roadside mowing is particularly important due to the high seed dispersal potential of mowing machines (Strykstra et al. 1997; Vitalos and Karrer 2009). To protect native plant communities, mowing before harvest, that is, in May or June, may be optimal.

## Author Contributions

**Henrietta Bak:** conceptualization (equal), data curation (equal), investigation (equal), methodology (equal), project administration (equal), writing – original draft (lead), writing – review and editing (equal). **Réka Fekete:** investigation (equal), writing – review and editing (equal). **Zsombor Miholcsa:** investigation (equal), methodology (equal), visualization (equal), writing – review and editing (equal). **Jenő Nagy:** formal analysis (equal), writing – review and editing (equal). **Sándor Jordán:** investigation (equal), writing – review and editing (equal). **Péter István Molnár:** writing – review and editing (equal). **Attila Molnár V.:** investigation (equal), writing – review and editing (equal). **Eszter Ruprecht:** conceptualization (equal), investigation (equal), methodology (equal), writing – original draft (supporting), writing – review and editing (equal).

## Conflicts of Interest

The authors declare no conflicts of interest.

## Supporting information


**Appendix S1:** ece372406‐sup‐0001‐AppendixS1.xlsx.

## Data Availability

The data that support the findings of this study are openly available in Supporting Information.
